# What affected Chinese parents’ decisions about tuberculosis (TB) treatment: Implications based on a cross-sectional survey

**DOI:** 10.1371/journal.pone.0245691

**Published:** 2021-01-25

**Authors:** Tingsong Xia, Juan Chen, Jian Rui, Jinxu Li, Yuli Guo

**Affiliations:** 1 Bao’an Center for Disease Control and Prevention, Shenzhen, Guangdong Province, China; 2 South China University of Technology, Shenzhen, China; The Chinese University of Hong Kong, HONG KONG

## Abstract

**Objective:**

Although progress has been made in tuberculosis (TB) treatment, China still remains one of the high-burden TB countries. One important reason that has not received sufficient scholarly attention is that Chinese individuals tend to underestimate the threat of TB. This contributed to the high rate of delay in seeking TB treatment and noncompliance with doctors’ regimen. Hence, this research examined how TB knowledge affected Chinese parents’ risk perceptions and their efficacy appraisal in TB treatment, and how their risk perception and efficacy appraisal affected their intentions to seek timely TB treatment for their children and adhere to doctors’ regimen.

**Methods:**

We conducted an online cross-sectional survey with 1129 parents of children attending kindergarten, primary school, and middle school in Shajing, a region with high TB incidence in China. Perceived severity of TB threat to self and to others, perceived susceptibility, response efficacy, and self-efficacy were measured, in addition to TB knowledge and intentions to seek timely TB treatment and adhere to doctors’ regimens.

**Results:**

Ordinal least squares regression demonstrated that TB knowledge was positively associated with perceived severity of TB threat to self, perceived severity of TB threat to others, perceived susceptibility, response efficacy, and self-efficacy, but it did not affect their medical decisions. In addition, binary logistic regression revealed that response efficacy and self-efficacy predicted both intentions positively, and perceived severity of TB threat to self only enhanced Chinese individuals’ intention to follow doctors’ regimens.

**Conclusion:**

Health education aimed at knowledge improvement may be effective in changing one’s perceptions of the given health threat but may not be effective to change their behavior. Thus, practitioners need to focus on changing Chinese parents’ perceptions of TB rather than simply improving their knowledge. Specifically, it is necessary to lower their efficacy in self-management and enhance their perceived infectiousness of TB.

## Introduction

TB remains a major public health threat worldwide. More than 10 million new TB cases were reported in 2019, causing 1.2 million deaths [1]. China reported the third largest number of TB patients in the world after India and Indonesia [1]. In 2019 there was an estimate of 833,000 new cases of TB in China, accounting for 8.4% of the total cases in the world [1]. As TB is more likely to break out in densely-populated environments such as schools [2], Chinese children can be vulnerable to this health threat.

Although TB can be cured if discovered early, China still remains among the 30 high-burden TB countries [1]. Two problems added to the severity of the spread of TB in China: the failure to seek timely TB treatment and comply with doctors’ regimens throughout the treatment. A meta-analysis of research on TB patients in China shows that only 47% of Chinese individuals with TB symptoms visited doctors timely and 59% adhered to doctors’ regimens throughout the process [3].

Previous research concluded that individuals’ failure to seek timely TB treatment and comply with doctors’ regimens was usually a result of health disparity [4,5]. However, specifically in China, another important reason may be that Chinese individuals tend to underestimate the threat of TB [6,7]. The progress in TB treatment made them perceive TB as less threatening and easy to cure [6,7]. Thus, the present research aims to investigate what factors shaped Chinese parents’ risk perception of TB and their efficacy appraisal in TB treatment. Another major question that the current study seeks to answer is how Chinese parents’ decisions about TB treatment, namely their intentions to seek timely TB treatment and comply with doctors’ regimens, varied as a function of their risk perception and efficacy appraisal.

Previous research conceptualized risk as a combination of perceived severity of negative outcomes and perceived likelihood of being affected by the given threat [8–10]. Given the high infectiousness of TB, we further distinguished perceived severity of TB threat to self and others. Thus, in this study, risk perception is conceptualized as the sum of three components: perceived severity of TB threat to self, perceived severity of TB threat to others, and perceived susceptibility. In addition, the extant research conceptualized efficacy appraisal as the sum or the average of response efficacy (one’s perception that the recommended act can solve the health problem) and self-efficacy (one’s confidence in performing the recommended act to avert the health threat) [8,10].

In the contemporary society, one major source of health information is media. Research shows that mediated health campaigns are effective in enhancing individuals’ health knowledge and could bridge the knowledge gap between different socioeconomic strata [11–13]. This knowledge can shape their risk perception and their appraisal of efficacy [14]. When it comes to TB, individuals with more knowledge are more likely aware that TB is an infectious disease that can cause adverse consequences but can be controlled and cured. In other words, high levels of TB knowledge should be related to high levels of risk perception and efficacy appraisal.

H1: TB knowledge is positively related to (a) perceived severity of TB threat to self, (b) perceived severity of TB threat to others, (c) perceived susceptibility, (d) response efficacy, and (e) self-efficacy.

These risk perceptions and efficacy appraisals can affect individuals’ engagement in health behavior. The effect of perceived severity of the threat and perceived likelihood on protective behavior has been well documented [15,16]. The innate aversion to potential loss prompts individuals to avoid health threats through protective behavior. Theoretical models like the health belief model [17], extended parallel process model [18], and risk perception attitude framework [15] suggest that individuals can be motivated to engage in protective behaviors if they think not performing the given acts can cause severe outcomes or heighten their chance to be affected. Indeed, meta-analyses demonstrated that perceived severity and perceived susceptibility could motivate individuals to adopt health behaviors [19–21].

In addition, high efficacy can change how individuals perceive the given health threat [22]. Those high in efficacy appraisal likely feel more hopeful that the given recommended acts are effective in averting the health threat [22] and more confident in overcoming barriers to performing these acts [23]. Thus, individuals reporting high response efficacy and high self-efficacy are more motivated to engage in the recommended protective behaviors [18,24,25].

H2: (a) Perceived severity of TB threat to self, (b) perceived severity of TB threat to others, (c) perceived susceptibility, (d) response efficacy, and (e) self-efficacy exhibited a positive effect on Chinese parents’ intention to seek timely TB treatment.
H3: (a) Perceived severity of TB threat to self, (b) perceived severity of TB threat to others, (c) perceived susceptibility, (d) response efficacy, and (e) self-efficacy exhibited a positive effect on Chinese parents’ intention to comply with TB treatment.

## Material and method

### Population and sampling

The Institutional Review Board at South China University of Technology approved this study. Participants were exposed to a page describing the purpose, procedure, risks, benefits, and contact information related to this study prior to the survey. They indicated their consent by clicking the button stating that they agreed to participate.

Our target population is parents of children attending kindergarten, elementary school, and middle school in Shajing. Until the end of 2018, Shajing had about 540,000 migrant workers out of 900,000 residents [26]. The high mobility of residents in Shajing increased the chance of infectious diseases like TB. Indeed, South Guangdong, where Shajing is located, is one of the six regions with high TB incidence in China [27]. We excluded high school students’ parents because most high school students in Shajing lived in school dorms. Thus, their parents’ influence on their health decisions may not be great compared to their teachers or themselves.

Shajing Health Inspection Institute (SHII) provided the sampling frame, which included 848 kindergarten classes, 1004 primary school classes, and 221 middle school classes. We estimated the number of sampled classes at each level of school (33 classes at kindergartens, 42 classes at primary schools, and nine classes at middle schools) by matching the proportion of classes at different levels of school. Next, we randomly selected classes from the list of schools. SHII then contacted parents at selected classes. However, not all parents were reached probably because many of them are migrant workers. Eventually, we received 1129 responses only from 27 classes at kindergartens, six classes at primary schools and five classes at middle schools.

### Procedure

Data collection lasted four weeks from August to September 2019. SHII distributed our online survey by sending its link to the parents at selected classes. As it was an online survey, we could not control the error during data collection. Therefore, trained research assistants completed data cleaning after data collection and dropped incomplete responses as well as repetitive data.

### Measures

We measured *the intention to seek timely TB treatment* by asking the participants to indicate the extent to which they agreed with the statement, “if my child is infected with TB, I will take him/her to see a doctor immediately” (1 = strongly disagree, 7 = strongly agree). A similar question was asked to measure *the intention to adhere to doctors’ regimen*, “if my child is infected with TB, I will make sure that I adhere to doctor’s treatment” (1 = strongly disagree, 7 = strongly agree).

We adapted the 7-point Likert scale that measures perceived severity, perceived susceptibility, response efficacy, and self-efficacy to measure relevant variables [28]. *Perceived severity of threat to self* was assessed with two items, “TB is a very serious disease” and “TB can cause pulmonary fibrosis and cavity, posing a serious threat to one’s health” (Cronbach’s *α* = .80). *Perceived severity of threat to others* was measured by two items, “TB is a serious infectious disease” and “TB is very infectious and will pose a serious threat to others” (Cronbach’s *α* = .88). *Perceived susceptibility* was measured through four items (e.g., “my child has a high risk of developing TB”; Cronbach’s *α* = .85). Three items were used to measure *response efficacy* (e.g., “modern medical knowledge and skills can prevent and cure TB effectively”; Cronbach’s *α* = .71). Two items were used to assess *self-efficacy*, including “I can master the knowledge of TB” and “I can prevent TB by engaging in the recommended behaviors” (Cronbach’s *α* = .82).

*TB knowledge* was measured with 17 questions based on the online manual about TB that Chinese Center for Disease Control and Prevention offered in March 2019 [29]. Participants received one point when they made one correct choice. This made the maximum score 67 points (Cronbach’s *α* = .80).

### Statistical analysis

Data analysis was completed through SPSS 23. Ordinal least squares (OLS) regression was conducted to test H1. Perceived severity of TB threat to self, perceived severity of TB threat to others, perceived susceptibility, response efficacy, and self-efficacy were entered the model separately as the dependent variable, whereas TB knowledge was treated as the independent variable. Although the dependent variables were measured on Likert scales, they can be treated as continuous variable [30–32]. Furthermore, the distribution of these dependent variables could be considered normal distribution, as their skewness (perceived severity of TB threat to self: -1.67, perceived severity of TB threat to others: -1.46, perceived susceptibility: .17, response efficacy: -1.35, self-efficacy: -1.62) and kurtosis (perceived severity of TB threat to self: 2.44, perceived severity of TB threat to others: 1.60, perceived susceptibility: -.42, response efficacy: 2.02, self-efficacy: 2.58) indicated. The assumption of multicollinearity was also met, as all variance inflation factor (VIFs) were between 1 and 2 (Table 1). Control variables included biological sex (0 = male, 1 = female), age, education (1 = no schooling, 2 = elementary school, 3 = middle school, 4 = high school, 5 = associate degree, 6 = bachelor’s degree, 7 = master’s or doctor’s degree), household monthly income (1 = 3500 RMB or less, 2 = 3501–5000 RMB, 3 = 5001–8000 RMB, 4 = 8001–12500 RMB, 5 = 12501–38500 RMB, 6 = 38501–83500 RMB, 7 = 83501 RMB or more), and the child’s grade (1 = kindergarten, 2 = elementary school, 3 = middle school). The interval of the household income was classified based on the household income in China provided by KPMG [33]. As many parents had more than one child, we asked parents to answer questions about the child at the class from which they received the invitation to the survey.

**Table 1:** The effect of TB knowledge on TB-related risk perception and efficacy appraisal.

	Perceived severity of TB threat to self	Perceived severity of TB threat to others	Perceived susceptibility	Response efficacy	Self-efficacy	VIF
TB knowledge	.10**	.13***	.15***	.27***	.31***	1.01
Biological sex (male as the reference group)	-.09**	-.03	-.10**	-.06*	-.04	1.02
Age	-.03	-.01	-.05	-.03	-.04	1.47
The child’s grade	.00	-.00	-.04	.04	.03	1.54
Education	-.02	.08**	.04	.01	.05	1.34
Income	-.01	-.04	-.05	.04	.02	1.29
Adjusted R^2^, F	.01**, 3.57	.02***, 4.62	.03***, 7.45	.07***, 15.60	.10***, 22.59	NA

Note.

* *p <* .*05*

** p < .*01*

***p < .*001*.

In addition, as the intention to seek timely TB treatment and the intention to comply with doctors’ regimens were highly skewed (see the frequency distribution of these two variables below), binary logistic regression was conducted to test H2 and H3. Responses indicating strong agreement with the statements about seeking timely TB treatment and complying with doctors’ regimens were coded as 1 and the rest as 0. Along with control variables mentioned above and TB knowledge, perceived severity of TB threat to self, perceived severity of TB threat to others, perceived susceptibility, response efficacy, and self-efficacy were all entered the model. Again, no evidence of multicollinearity was discovered, as all VIFs were between 1 and 2 (Table 2).

**Table 2:** The effect of TB-related risk perception and efficacy appraisal on Chinese parents’ decisions about TB treatment; B, odds ratio and 95% confidence interval.

	Seeking Timely TB Treatment				Compliance			
	B	Odds ratio	95% CI	VIF	B	Odds ratio	95% CI	VIF
Perceived severity of TB threat to self	.21	1.24	[.94, 1.63]	1.67	.36**	1.43	[1.14, 1.80]	1.67
Perceived severity of TB threat to others	.15	1.16	[.90, 1.49]	1.80	.13	1.14	[.91, 1.42]	1.80
Perceived susceptibility	-.22	.81	[.61, 1.06]	1.16	-.08	.92	[.73, 1.16]	1.16
Response efficacy	.75***	2.12	[1.48, 3.03]	1.63	.45**	1.57	[1.15, 2.15]	1.63
Self-efficacy	.95***	2.57	[1.80, 3.69]	1.69	.77***	2.16	[1.58, 2.97]	1.69
TB knowledge	.02	1.02	[1.00, 1.05]	1.15	.02	1.02	[.99, 1.04]	1.15
Biological sex (male as the reference group)	.62	1.85	[.93, 3.70]	1.04	.56	1.76	[.97, 3.19]	1.04
Age	.03	1.03	[.97, 1.09]	1.48	.02	1.02	[.97, 1.08]	1.48
The child’s grade	-.18	.83	[.52, 1.33]	1.54	-.31	.74	[.49, 1.10]	1.54
Income	-.03	.97	[.73, 1.28]	1.30	-.06	.95	[.75, 1.20]	1.30
Education	.28	1.33	[.95, 1.85]	1.35	.27	1.31	[.97, 1.76]	1.35
χ^2^, df, pseudo R^2^ (Cox & Snell R^2^, Nagelkerke R^2^)	χ^2^(11) = 181.30***, .15, .43				χ^2^(11) = 156.22***, .13, .34			

Note.

** p < .*01*

***p < .*001*.

## Results and discussion

### Descriptive statistics

Our final sample had more females (71.6%) than males (28.4%). The average age was almost 35 years old (*M* = 34.9, *SD* = 5.82). The majority of the participants completed high school (32.2%) or middle school (31.6%), followed by associate degree (22.4%), college (8.9%), and master/PhD (.4%). Thus, less than 10% of our participants completed college education. In terms of their financial condition, about 27.5% of our participants reported their household monthly income of 8001–12500 RMB ($1119.48-$1748.97), 22.7% with 5001–8000 RMB ($699.73-$1119.34), 22.3% with 12501–38500 RMB ($1749.11-$5386.82), 17.4% with 3501–5000 RMB ($489.85-$699.59), 5.9% with 3500 RMB or less ($489.71 or less), 3.0% with 38501–83500 RMB ($5386.96-$11683.11), and 1.2% earned more than 83501 RMB ($11683.25 or higher).

Overall, our participants reported very high levels of intention to seek timely TB treatment (*M* = 6.88, *SD* = .57) and comply with doctors’ regimens (*M* = 6.86, *SD* = .61). Most participants indicated strong agreement with the statements about seeking timely TB treatment (94.5%), followed by moderate agreement (2.1%), slight disagreement (1.9%), neutral stance (1.2%), and strong disagreement (.3%). Similarly, the majority of respondents indicated strong agreement with the statements about complying with doctors’ regimens on TB treatment (93.4%), followed by moderate agreement (3.0%), neutral stance (1.8%), slight disagreement (1.2%), strong disagreement (.4%), and moderate disagreement (.2%).

Next, our participants reported high levels of perceived severity of TB threat to self (*M* = 6.16, *SD* = 1.28), perceived severity of TB threat to others (*M* = 5.99, *SD* = 1.40), response efficacy (*M* = 6.22, *SD* = .93), and self-efficacy (*M* = 6.40, *SD* = .91). However, their average level of perceived susceptibility was medium (*M* = 4.09, *SD* = 1.52).

Finally, the current level of TB knowledge that our participants demonstrated is not promising. Recall that the maximum possible point was 67. While the highest score was 66, the lowest was only 5. Thus, our participants demonstrated a wide range of TB knowledge. In addition, the average score was 41.5, which was equal to 61.9%, with a medium of 44 (65.67%) and a standard deviation of 11.59. Twelve participants (1.1%) answered 90% of questions correctly, with 12.4% scoring between 80% and 89.9%, 24.9% scoring between 70% and 79.9%, 22.1% scoring between 60% and 69.9%, and 39.5% scoring below 60%.

### Hypotheses testing

Table 1 presents results of how TB knowledge predicted TB-related risk perception and efficacy appraisal. TB knowledge exhibited a positive effect on perceived severity of TB threat to self (*β* = .10, *p* < .01), perceived severity of TB threat to others (*β* = .13, *p* < .001), perceived susceptibility to TB (*β* = .15, *p* < .001), response efficacy in TB treatment (*β* = .27, *p* < .001), and self-efficacy in TB treatment (*β* = .31, *p* < .001). Thus, H1a through e were all supported.

Table 2 presents results of how TB-related risk perception and efficacy appraisal predicted Chinese parents’ intention to seek timely treatment and comply with doctors’ regimens. Response efficacy (B = .75, OR = 2.12, 95% CI: [1.48, 3.03], *p* < .001), and self-efficacy (B = .95, OR = 2.57, 95% CI: [1.80, 3.69], *p* < .001) predicted Chinese parents’ intention to seek timely TB treatment positively.

In addition, perceived severity of TB threat to self (B = .36, OR = 1.43, 95% CI: [1.14, 1.80], *p* < .01), response efficacy (B = .45, OR = 1.57, 95% CI: [1.15, 2.15], *p* < .01), and self-efficacy (B = .77, OR = 2.16, 95% CI: [1.58, 2.97], *p* < .001) predicted Chinese parents’ intention to comply with doctors’ regimens positively. Therefore, for both H2 and H3, hypotheses d and e were supported, but b and c were rejected. H3a also received support.

Finally, we conducted power analysis through G*Power 3.1.9.7. For power analysis on H1, we chose linear multiple regression with fixed model and R^2^ increase as the statistical test we conducted. In order to complete power analysis, G*Power requires users to provide effect size, α, total sample size, the number of tested predictors, and total number of predictors. Except the effect size, all the other parameters are self-explanatory. We calculated the effect size based on partial R^2^. The power of all five OLS regressions was .92 (perceived severity of TB threat to self), .99 (perceived severity of TB threat to others), 1.00 (perceived susceptibility), 1.00 (response efficacy), and 1.00 (self-efficacy) consecutively. Therefore, the current OLS regressions achieved satisfactory power.

Additionally, the power analysis for two binary logistic regression models was also conducted. In order to complete the power analysis for binary logistic regression, G*Power requires users to enter odds ratio, P_r_ (Y = 1) H_0_, α, total sample size, R^2^ other X, X distribution, X parm μ, and X parm σ. Except odds ratio and P_r_ (Y = 1) H_0,_ all the other parameters were either self-explanatory or automatically set up. Following the manual about G*Power [34], we set P_r_ (Y = 1) H_0_ as .5. As mentioned earlier, 94.5% of participants expressed strong agreement with seeking timely TB treatment and 93.4% of participants expressed strong agreement with complying with doctors’ regimens. We thus used these two numbers to calculate odds ratio. Finally, according to G*Power, the power of the two binary logistic regression models was 1.

### Discussion of major findings

Parents usually make medical decisions for children. Therefore, understanding what factors affected parents’ decisions on TB treatment can be critical to children’s health. Previous research suggests that the progress in TB control in China made Chinese individuals underestimate the level of threat of TB and develop an optimistic attitude towards curing TB [6,7]. This might explain why a large number of TB patients in China delayed in their seeking for TB treatment and did not comply with doctors’ regimens [6,7]. Thus, understanding how Chinese parents perceive the threat level of TB and evaluate their confidence in controlling TB can be important, as their risk perception and efficacy appraisal may predict their intention to seek timely TB treatment and comply with doctors’ regimens. Furthermore, this investigation could provide important implications on future campaigns that aim to influence Chinese parents’ decisions on TB treatment.

Our descriptive statistics shows that most of the participants expressed strong intentions to seek timely treatment for TB and comply with doctors’ recommendations on TB treatment. However, it is important to note that we examined their behavioral intention rather than actual behavior. In other words, our participants might not necessarily translate their strong intention into behavior. This speculation is not only a result of the intention-behavior gap but also can be derived from their high levels of response efficacy and self-efficacy in TB treatment. Consistent with previous research [6,7], the present research provided additional evidence which demonstrates Chinese individuals’ optimism in controlling TB. Over-confidence can make individuals underestimate the barriers to performing the given act and increase the chance of errors [35]. Therefore, it is necessary to lower Chinese parents’ self-efficacy in self-management of TB. However, self-efficacy in seeking and following professional advice should be maintained and enhanced by reducing the behavioral barriers of our target population.

When it comes to risk perception, Chinese parents reported the highest level of perceived severity of TB threat to self, followed by perceived severity of TB threat to others and perceived susceptibility. Post-hoc analysis on dependent sample t-test shows that their differences were statistically significant. Hence, Chinese parents seemed to think TB poses a severe threat to their own health but might not to others. One possible explanation is that our participants might lack knowledge of the infectiousness of TB. For instance, one set of eight questions asked how one can be infected with TB. Only 2% of participants answered all questions correctly. Moreover, only half of the participants knew that quarantine was necessary if their children (correct rate: 48.4%) or other children (correct rate: 48%) were infected with TB. Hence, future campaigns on this population should highlight the infectiousness of TB and emphasize its threat to *all* children. For example, campaign messages could provide information about TB transmission, emphasize its high likelihood to spread in densely-populated environments, and explain why children can have a relatively high likelihood of being infected with TB. Protective measures that control TB spread are also needed in future education.

In addition, our results explicated how Chinese parents developed their risk perception of TB and evaluated the efficacy level of TB treatment. The current findings provided consistent evidence demonstrating positive associations between TB knowledge and all five dimensions of risk perception and efficacy appraisal. In other words, individuals that exhibited more knowledge were inclined to perceive TB more severe, view them more subject to TB, and express more confidence in TB treatment and performing preventive measures against TB. This might be because people knowing more about TB tended to develop more precise understanding of this disease. However, TB knowledge was not significantly related to Chinese parents’ intention to seek timely TB treatment and comply with doctors’ regimens. Taken together, these findings suggest that health education aimed at knowledge improvement may be able to change individuals’ perceptions but may not be effective in affecting their behavior directly. Furthermore, this also provides implications on the role that health knowledge plays in health campaigns. If behavior change is the ultimate goal, the key may be to influence how our target audience perceives certain health risks. However, if the ultimate goal is to change the target audience’s perceptions of certain health threats, the focus of the campaign should be information provision.

Furthermore, we found that response efficacy and self-efficacy predicted Chinese parents’ intention to visit doctors timely and adhere to doctors’ regimen. These findings reveal the great impact of efficacy appraisal on taking preventive actions against TB. Moreover, compared to perceived severity of TB threat to self, response efficacy and self-efficacy exhibited a greater effect on Chinese parents’ decisions on TB treatment. This aligns with prior research which posited that fear appeal is not sufficient for behavior change; enhancing the confidence level of the target audience is more important than making them scared [24,25]. However, as explained earlier, our participants already reported high levels of response efficacy and self-efficacy, which could cause a boomerang effect. This suggests that future campaigns need to distinguish self-efficacy in self-management and self-efficacy in following professional advice. While the former needs to be kept at a minimum, the latter needs to be enhanced by lowering the target audience’s barriers to performing recommended acts. In addition, the nonsignificant effects of perceived severity of threat to others and perceived susceptibility might be because our participants lack knowledge of the infectiousness of TB and thereby underestimated their susceptibility to TB and the severity of its threat to others, making the effects of these variables nonsignificant.

### Limitations and future directions

Although the present investigation provides implications for relevant research and campaigns on TB treatment in China, several limitations are warrant discussions. Fist, this study is conducted in China and limited to one health threat. These could limit the generalizability of the current findings. Comparisons between different health threats, populations, and cultures are needed.

Second, most of our participants reported lower levels of education and income. This of course could be a threat to the validity of our findings. However, previous research shows that the delay in seeking timely TB treatment and failure to comply with doctors’ regimen happened more among people of low socioeconomic statuses [3].

Third, the cross-sectional nature of this study inhibits us from making causal arguments. For example, individuals perceiving TB as a severe threat may be more motivated to seek information about TB, resulting in an enhanced level of TB knowledge. Longitudinal research with panel designs can address this limitation.

Additionally, we measured parents’ intention to visit doctors timely and adhere to doctors’ regimens with only one item. This could threaten the validity of our measures.

Next, as explained earlier, many parents could not be reached. Thus, the final sample was much smaller, which could threaten the representativeness of the sample.

Finally, as mentioned above, we measured behavioral intention. The gap between behavioral intention and actual behavior in TB treatment can result from multiple factors such as one’s financial condition.

In addition to addressing these limitations mentioned above, future research can further examine how individuals develop their TB-related risk perception and efficacy appraisal. The channels through which they seek health information can be a potential predictor. Demographic variables such as age, education, and income can affect individuals’ choices of health information channels, which may provide additional insights on knowledge gap.

Other variables that might influence the outcome variables should also be considered in future research. For instance, whether individuals have been infected with TB can affect their TB knowledge and their experience of TB consultation.

Future research should also investigate how Chinese individuals weigh the benefits and costs of seeking timely TB treatment and following doctors’ regimens. Prior research found that Chinese students were reluctant to visit doctors for TB because they were afraid that the treatment could affect their study [7]. Hence, benefits and costs associated with TB treatment may not be limited to health-relevant variables. Investigations on these factors can not only improve our understanding of how Chinese parents make decisions on TB treatment for their children but provide more implications on TB campaigns.

## Conclusion

Knowledge was positively related to Chinese parents’ TB-related risk perception and efficacy appraisal. Chinese parents’ intention to seek timely TB treatment and comply with doctors’ regimens was a function of perceived severity of TB threat to self, response efficacy, and self-efficacy, but not TB knowledge. These results suggest that when it comes to changing Chinese parents’ decisions on TB treatment, it may be necessary to focus on their perception rather than improve their knowledge. Specifically, lowering their efficacy to a moderate level and enhancing their perceived infectiousness of TB may be important.

## Endnote

We did not provide the justification for the sample size of the current study because this paper is part of a larger baseline project on Chinese parents’ perception and knowledge of tuberculosis. Therefore, we conducted sample size calculation based on other statistical analyses published in another study. As an alternative to sample size calculation, we conducted power analysis to demonstrate that our sample size should be sufficient.

## Supporting information

S1 Dataset (SAV)

S1 File (DOCX)

S2 File (DOCX)
