# Supplementary material for: What affected Chinese parents’ decisions about tuberculosis (TB) treatment: Implications based on a cross-sectional survey
Source: PLoS One. 2021 Jan 25;16(1):e0245691. doi: 10.1371/journal.pone.0245691 (PMC7833143; doi:10.1371/journal.pone.0245691)
Supplement: S1 File — (DOCX) [file pone.0245691.s002.docx]

1. 肺结核是一种非常严重的疾病。

1=完全反对

2=大部分反对

3=有点反对

4=中立

5=有点赞同

6=大部分赞同

7=完全赞同

1. 肺结核会造成肺部纤维化甚至肺部空洞，对健康造成严重威胁。

1=完全反对

2=大部分反对

3=有点反对

4=中立

5=有点赞同

6=大部分赞同

7=完全赞同

1. 肺结核是一种严重的传染病。

1=完全反对

2=大部分反对

3=有点反对

4=中立

5=有点赞同

6=大部分赞同

7=完全赞同

1. 肺结核的传染性很强，会对别人造成严重威胁。

1=完全反对

2=大部分反对

3=有点反对

4=中立

5=有点赞同

6=大部分赞同

7=完全赞同

1. 我的孩子患肺结核的风险很高。（注：如果您有一个孩子请按照您收到该问卷的所在班级的孩子回答问题）

1=完全反对

2=大部分反对

3=有点反对

4=中立

5=有点赞同

6=大部分赞同

7=完全赞同

1. 学生群体患肺结核的机率很大。

1=完全反对

2=大部分反对

3=有点反对

4=中立

5=有点赞同

6=大部分赞同

7=完全赞同

1. 我的孩子有可能被他人传染肺结核。

1=完全反对

2=大部分反对

3=有点反对

4=中立

5=有点赞同

6=大部分赞同

7=完全赞同

1. 学生群体被他人传染肺结核的概率很高。

1=完全反对

2=大部分反对

3=有点反对

4=中立

5=有点赞同

6=大部分赞同

7=完全赞同

1. 了解肺结核相关知识，能有效预防肺结核。

1=完全反对

2=大部分反对

3=有点反对

4=中立

5=有点赞同

6=大部分赞同

7=完全赞同

1. 即使感染了肺结核，只要及时就医谨遵医嘱，肺结核完全可以治愈。

1=完全反对

2=大部分反对

3=有点反对

4=中立

5=有点赞同

6=大部分赞同

7=完全赞同

1. 现代医学知识和技术可以有效预防和治疗肺结核。

1=完全反对

2=大部分反对

3=有点反对

4=中立

5=有点赞同

6=大部分赞同

7=完全赞同

1. 我可以掌握肺结核的相关知识。

1=完全反对

2=大部分反对

3=有点反对

4=中立

5=有点赞同

6=大部分赞同

7=完全赞同

1. 我能遵照医生的建议，有效预防肺结核。

1=完全反对

2=大部分反对

3=有点反对

4=中立

5=有点赞同

6=大部分赞同

7=完全赞同

1. 如果我的孩子感染了肺结核，我会带他去及时就医。

1=完全反对

2=大部分反对

3=有点反对

4=中立

5=有点赞同

6=大部分赞同

7=完全赞同

1. 如果我的孩子感染了肺结核，我会确保他严格遵照医嘱治疗。

1=完全反对

2=大部分反对

3=有点反对

4=中立

5=有点赞同

6=大部分赞同

7=完全赞同

1. 如果我的孩子得了肺结核，我会感到自卑。

1=完全反对

2=大部分反对

3=有点反对

4=中立

5=有点赞同

6=大部分赞同

7=完全赞同

1. 如果我的孩子得了肺结核，我会感到恐慌。

1=完全反对

2=大部分反对

3=有点反对

4=中立

5=有点赞同

6=大部分赞同

7=完全赞同

1. 如果我的孩子得了肺结核，这会影响到别人对他的看法。

1=完全反对

2=大部分反对

3=有点反对

4=中立

5=有点赞同

6=大部分赞同

7=完全赞同

1. 如果我的孩子得了肺结核，他可能会被同学孤立。

1=完全反对

2=大部分反对

3=有点反对

4=中立

5=有点赞同

6=大部分赞同

7=完全赞同

1. 如果我的孩子出现了咳嗽、咳痰、不明原因的低热、入睡后出汗异常、乏力、消瘦等症状时，此时应该___________________。请选择你认为正确的所有选项。
2. 警惕是不是感染了肺结核
3. 及时请假
4. 报告老师或校医
5. 主动隔离
6. 尽快就医
7. 我觉得他可以扛得住，应该继续正常上学、生活
8. 如果我的孩子班上有同学出现了咳嗽、咳痰、不明原因的低热、入睡后出汗异常、乏力、消瘦等症状时，此时应该___________________。请选择你认为正确的所有选项。
9. 警惕他是不是感染了肺结核
10. 建议他及时请假
11. 报告老师或通知校医
12. 建议他主动隔离
13. 建议他尽快就医
14. 我觉得他可以扛得住，应该继续正常上学、生活

**请您务必如实回答以下问题。如果不知道就选不知道**

1. 结核病是可以预防的。
2. 正确
3. 错误
4. 不知道
5. 判断：结核病是可以治愈的。
6. 正确
7. 错误
8. 不知道
9. 结核病由__________引起。此题为单选。
10. 结核分枝杆菌
11. 肺炎球菌
12. 结核球菌
13. 链球菌
14. 不知道
15. 结核病可以发生在什么部位？请选择您认为正确的所有选项。
16. 肺部
17. 脑部
18. 胸部
19. 皮肤
20. 牙齿
21. 头发
22. 不知道
23. 《中华人民共和国传染病防治法》将常见传染病分为甲乙丙三个等级。下列传染病分属哪个等级？请在您认为属于甲类传染病的疾病后面写上1，乙类传染病的疾病后面写上2，丙类传染病的疾病后面写上3。
24. 艾滋病
25. 肺结核
26. 鼠疫
27. 非典
28. 流感
29. 霍乱
30. 麻风病
31. 不知道
32. 肺结核有什么常见症状？请选择你认为正确的所有选项。
33. 咳嗽、咳痰、咯血、胸疼、呼吸困难
34. 午后低热、面颊潮红、入睡后出汗异常、乏力
35. 食欲不振、体重下降、女性月经失调
36. 少数患者会出现结节性红斑、皮下结节、疱疹性结膜炎、结合风湿病
37. 不知道
38. 肺结核的传染途径有哪些？请选择您认为正确的所有选项。
39. 患病者咳嗽、打喷嚏
40. 患病者随地吐痰
41. 和肺结核病人握手
42. 和肺结核病人共用餐具
43. 吃肺结核病人吃剩下来的东西
44. 喝未经消毒的牛奶或乳制品
45. 坐肺结核病人坐过的椅子
46. 肺结核病人的血液也有传染性
47. 不知道
48. 哪些人是肺结核的高发人群？请选择您认为正确的所有选项。
49. 体弱多病者
50. 老年人
51. 糖尿病人
52. 艾滋病人
53. 经常抽烟的人
54. 因患有慢性病或长期服用激素类药物而导致免疫力下降的人
55. 不知道
56. 哪些行为可以预防肺结核？请选择您认为正确的所有选项。
57. 房间多通风
58. 到结核病人聚集场所要戴口罩
59. 不随地吐痰
60. 不朝着他人打喷嚏
61. 保证营养，戒烟戒酒，睡眠充足，加强锻炼
62. 定期体检
63. 预防和控制其他相关疾病
64. 不知道
65. 肺结核患者的饮食应该注意哪些？请选择您认为正确的所有选项。
66. 高热量食物
67. 高蛋白食物
68. 高维生素
69. 多膳食纤维和水
70. 不知道
71. 如果感染了肺结核应该怎么办？请选择您认为正确的所有选项。
72. 及时就医，及时复诊
73. 谨遵医嘱，按时服药，按疗程服药
74. 适当锻炼，劳逸结合，保持乐观情绪
75. 戒烟戒酒
76. 注意饮食，补充营养
77. 不知道
78. 如果家中有肺结核患者，怎样可以避免传染？请选择您认为正确的所有选项。
79. 最好让病人单独住一间房，至少要让他单独睡一张床
80. 病人居住的房间要保持空气流通，阳光充足
81. 病人要坚持戴口罩，不要亲近婴幼儿
82. 病人不要对着别人面部讲话、咳嗽、打喷嚏
83. 要对病人的餐具、便器、痰盂等物品进行消毒处理
84. 病人不能随地吐痰，而要把痰吐到有消毒剂的有盖容器中
85. 不知道
86. 在所有结核病中，仅有排菌期的肺结核患者才具有传染性。
87. 正确
88. 错误
89. 不知道
90. 一旦感染了结核菌，一定会发病。
91. 正确
92. 错误
93. 不知道
94. 接种过卡介苗就一定不会得结核病。
95. 正确
96. 错误

C. 不知道

**我们向您郑重承诺，您的所有回答都不会被用于商业用途。**

1. 您的性别是：
2. 男
3. 女
4. 您今年_______岁。
5. 您的孩子是_______。
6. 男孩
7. 女孩
8. 您的孩子今年_______岁。
9. 您的孩子今年几年级？
10. 您目前的职业状态是？
11. 无业
12. 机关事业单位
13. 企业单位
14. 农民
15. 其它
16. 您的配偶目前的职业状态是？
17. 无业
18. 机关事业单位
19. 企业单位
20. 农民
21. 其它
22. 您获得的最高学历是？
23. 未接受过学校教育
24. 小学
25. 初中
26. 高中
27. 大专
28. 本科
29. 硕士、博士
30. 您的配偶获得的最高学历是？
31. 未接受过学校教育
32. 小学
33. 初中
34. 高中
35. 大专
36. 本科
37. 硕士、博士
38. 您有几个孩子？
39. 您的家庭月收入？
40. 等于或低于3500元
41. 3501-5000元
42. 5001-8000元
43. 8001-12500元
44. 12501-38500元
45. 38501-83500元
46. 83501元及以上
47. 请评估一下您的孩子的健康状况。

1=非常不健康

2=不健康

3=一般

4=比较健康

5=非常健康
