# Supplementary material for: What affected Chinese parents’ decisions about tuberculosis (TB) treatment: Implications based on a cross-sectional survey
Source: PLoS One. 2021 Jan 25;16(1):e0245691. doi: 10.1371/journal.pone.0245691 (PMC7833143; doi:10.1371/journal.pone.0245691)
Supplement: S2 File — (DOCX) [file pone.0245691.s003.docx]

Please answer questions 1-19 by using the following scale.

1=strongly disagree

2=somewhat disagree

3=slightly disagree

4=neither disagree nor agree

5=slightly agree

6=somewhat agree

7=strongly agree

1. Tuberculosis is a very serious disease.
2. Tuberculosis can cause fibrotic fibrosis and even cavity in the lung, which poses a severe threat to health.
3. Tuberculosis is a severe infectious disease.
4. With a high level of infectiousness, tuberculosis can threaten others seriously.
5. My child has a high level of being infected with tuberculosis. If you have more than one child please answer the question about the child from whose class you received this invitation.
6. Students have a high chance of getting tuberculosis.
7. My child can be infected with tuberculosis from others.
8. Students have a high chance of being infected with tuberculosis.
9. Getting knowledge of tuberculosis can prevent this disease effectively.
10. Even if someone gets tuberculosis, as long as they visit doctors timely and follow doctors’ recommendations, they can be cured completely.
11. modern medical knowledge and skills can prevent and cure TB effectively.
12. I can master the knowledge of tuberculosis through learning.
13. I can prevent tuberculosis by engaging in the recommended behavior by doctors.
14. If my child gets tuberculosis, I will take him/her to doctors immediately.
15. If my child gets tuberculosis, I will make sure that I adhere to doctors’ treatment.
16. If my child gets tuberculosis, I will feel shamed.
17. If my child gets tuberculosis, I will feel panicked.
18. If my child gets tuberculosis, it will influence how he/she is viewed.
19. If my child gets tuberculosis, he/she may be isolated at school.
20. If my child coughs with phlegm, unusual fever, sweats after sleeping, and loses weight with fatigue, I should __________. Please select all the options that you think are correct.
21. Be cautious if he/she gets tuberculosis.
22. Ask for medical leave immediately
23. Report to the teacher or the school doctor.
24. Keep him/her quarantine
25. Take him/her to the doctor immediately.
26. Think he/she can handle it and continue to school.
27. If a child at my child’s class coughs with phlegm, unusual fever, sweats after sleeping, and loses weight with fatigue, I should __________. Please select all the options that you think are correct.
28. Be cautious if he/she gets tuberculosis.
29. Ask for medical leave immediately
30. Report to the teacher or the school doctor.
31. Keep him/her quarantine
32. Take him/her to the doctor immediately.
33. Think he/she can handle it and continue to school.
34. Tuberculosis can be prevented.
35. True
36. False
37. I don’t know
38. Tuberculosis can be cured.
39. True
40. False
41. I don’t know
42. What causes tuberculosis? There is only one correct answer to the question.
43. Mycobacterium tuberculosis
44. Pneumoniae
45. Coccus
46. Streptococcus
47. I don’t know
48. Which part can be infected with tuberculosis? Please select all choices that you think are correct.
49. Lung
50. Brain
51. Breast
52. Skin
53. Teeth
54. Hair
55. I don’t know
56. According to the Law of Infectious Disease Prevention in People’s Republic of China, infectious diseases are classified into three levels: A, B, C. Which level do the following infectious diseases belong to? Please write 1 after the disease you think is A level, 2 after the disease you think is B level, and 3 after the disease you think is C level.
57. HIV/AIDS: A, B, C, I don’t know
58. Tuberculosis: A, B, C, I don’t know
59. Plague: A, B, C, I don’t know
60. SARS: A, B, C, I don’t know
61. Influenza: A, B, C, I don’t know
62. Cholera: A, B, C, I don’t know
63. Leprosy: A, B, C, I don’t know
64. What are common symptoms of tuberculosis? Please select all choices that you think are correct.
65. Cough, with phlegm or blood, pain in breast, hard to breath
66. Fever in the afternoon, flushing of cheeks, sweat after sleeping, fatigue
67. Loss of appetite and weight, menstrual disorder
68. Some patients can have erythema nodosum, subcutaneous nodules, herpetic conjunctivitis, and rheumatism.
69. I don’t know
70. How can you get tuberculosis? Please select all choices that you think are correct.
71. Patients cough and sneeze
72. Patients spit
73. Shake hands with patients
74. Share dinner sets with patients
75. Eat the leftover by patients
76. Drink milk that is not sanitized
77. Take the seat that patients have taken
78. Patients’ blood is also infectious
79. I don’t know
80. Who are vulnerable to tuberculosis? Please select all choices that you think are correct.
81. Those that are weak
82. Seniors
83. Diabetic patients
84. People with HIV/AIDS
85. Heavy smokers
86. Those with a weak immune system because they have chronic diseases or have taken hormonal drugs
87. I don’t know
88. What behaviors can prevent tuberculosis? Please select all choices that you think are correct.
89. Keep the room with air
90. Wear a facemask when you go to a place where there are tuberculosis patients
91. Don’t spit
92. Don’t sneeze at others’ face
93. Eat nutritious food. Don’t smoke or drink. Sleep well. Take exercises.
94. Take physical examination regularly
95. Prevent and control other diseases.
96. I don’t know
97. What food should tuberculosis patients eat? Please select all choices that you think are correct.
98. Diet with high levels of calorie
99. Diet with high levels of protein
100. Diet with high levels of vitamins
101. Die with high levels of fibers and water
102. I don’t know
103. What should you do if infected with tuberculosis? Please select all choices that you think are correct.
104. Seek timely treatment with timely follow-ups
105. Adhere to doctors’ regimens. Take medicine on time and according to the course of treatment
106. Exercise moderately. Keep a balance between work and rest. Be optimistic.
107. Quit drinking and smoking.
108. Make sure you eat food with good nutrition. Make up what you need.
109. I don’t know
110. What can be done to prevent being infected with there are tuberculosis patients at home? Please select all choices that you think are correct.
111. It’s better to give the patient a separate room. At least make a separate bed for him/her.
112. The room where the patient stays should be kept with enough air and sunshine.
113. The patient should keep wearing a facemask and avoid getting close contact with babies and children.
114. The patient should not talk, cough or sneeze at others’ face.
115. Sanitize the tableware, toilet, and spittoon used by patients.
116. The patient shouldn’t spit to the floor or ground but the container with a cap.
117. I don’t know.
118. Only tuberculosis with discharge of bacteria can be infectious.
119. True
120. False
121. I don’t know
122. Tuberculosis will definitely become active if someone is infected.
123. True
124. False
125. I don’t know
126. If you take the BCG vaccine, you will not get tuberculosis.
127. True
128. False
129. I don’t know
130. What’s your biological sex?
131. Male
132. Female
133. How old are you?
134. Your child is _______. If you have more than one child please answer the question about the child from whose class you received this invitation.
135. Boy
136. Girl
137. How old is your child?
138. What grade is your child?
139. What is your current occupation?
140. Unemployed
141. State-owned organization
142. Private sector
143. Peasants
144. Other
145. What is your spouse’s current occupation?
146. Unemployed
147. State-owned organization
148. Private sector
149. Peasants
150. Other
151. What’s the highest education degree you have received?
152. No schooling
153. Elementary school
154. Middle school
155. High school
156. Associate degree
157. Undergraduate degree
158. Master’s degree or Ph.D.
159. What’s the highest education degree your spouse has received?
160. No schooling
161. Elementary school
162. Middle school
163. High school
164. Associate degree
165. Undergraduate degree
166. Master’s degree or Ph.D.
167. How many children do you have?
168. What’s your monthly household income?
169. 3500 RMB or less
170. 3501-5000 RMB
171. 5001-8000 RMB
172. 8001-12500 RMB
173. 12501-38500 RMB
174. 38501-83500 RMB
175. 83501 or more
176. Please rate the health condition of your child.
177. Very unhealthy
178. Unhealthy
179. Neither unhealthy nor healthy
180. Healthy
181. Very healthy
